# SpecAtt-Net: Time-Frequency-Based Image Representations of Slice-Level Radiomic Features for Explainable Lung Cancer Classification via Slice-Wise Activation Mapping

**DOI:** 10.3390/diagnostics16152436

**Published:** 2026-08-01

**Authors:** Merve Ceyhan, Uğur Gürel

**Affiliations:** Department of Computer Engineering, Faculty of Engineering and Architecture, Eskişehir Osmangazi University, Odunpazarı, Eskişehir 26040, Turkey; ugurel@ogu.edu.tr

**Keywords:** cancer, lung cancer, radiomic, short-time Fourier transform, feature maps, classification, deep learning, slice labeling, explainability, clinical decision support

## Abstract

**Background**: Extracting radiomic features from volumetric imaging data for lung cancer classification is limited by high dimensionality and the black-box nature of deep learning models. Traditional methods may overlook dependencies between slices, leading to the loss of sequential spatial information. Even when classification is correct, the model’s explanatory power may remain insufficient. **Objective**: This study proposes SpecAtt-Net, which processes spectral representations of slice-level radiomic data from computed tomography (CT) scans of lung cancer patients. A slice-based explainability approach is also presented to identify the slice groups used in model decision-making. **Methods**: Slice-level radiomic data were preprocessed, and slice-position–frequency representations were generated using the Short-Time Fourier Transform (STFT). This enables the model to capture spectral textures and spatial transitions from 720 × 288 feature maps. SpecAtt-Net employs a dual-attention mechanism to focus on distinctive radiomic features. To address the lack of slice-level labels, Slice-Wise Activation Mapping (SWAM), a post hoc interpretability technique, was developed. SWAM converts two-dimensional (2D) activation maps into one-dimensional (1D) importance vectors and provides a weakly supervised indication of the slice groups most relevant to the model’s decision. **Results**: SpecAtt-Net achieved competitive performance in accuracy, precision, recall, and F1-score compared with the evaluated reference architectures. SWAM highlighted slice-sequence regions contributing strongly to model predictions, providing preliminary evidence for interpretability. **Conclusions**: SpecAtt-Net provides a compact and interpretable approach for lung cancer subtype classification using attention-based feature modeling. SWAM offers an exploratory visualization of decision-relevant sequence regions and may support future radiological interpretation and validation studies.

## 1. Introduction

In modern oncology, diagnosis, prognostic assessment, and treatment response status are very important issues [1,2,3]. Today, radiomic features extracted from imaging data enable numerical characterization of cancer tissue heterogeneity, its biological behavior, and the structural complexities surrounding the tumor [4,5]. Radiomic data consist of complex numerical data obtained from different modalities, which are multidimensional and do not change over time [6]. Signal processing methods that analyze such numerical data are needed to reveal their structure, frequencies, and similarities. The Short-Time Fourier Transform (STFT), a technique that evaluates how a signal’s frequency content changes over time, is one of the most widely used signal processing methods [7,8]. STFT enables the reconstruction of radiomic data in the time-frequency domain. By separating the numerical features extracted from cancer images into regional windows, STFT can reveal both the local frequency content and the structural state of tumor-specific variations. Thus, the one-dimensional structure of classical radiomic features can be transformed into a two-dimensional time-frequency map with STFT. With this transformation, important biological data such as cancer heterogeneity, tumor aggressiveness, and treatment response dynamics become more visible and analyzable [9]. Recent studies have shown that artificial intelligence models perform well on radiomic data, highlighting the need for a more qualitative representation of the data [10]. STFT’s ability to convert radiomic features into images enables the use of image-based deep learning methods, especially convolutional neural networks. This helps overcome many of the limitations encountered in radiomic analysis. With this method, rich representations with discriminatory power can be produced while preserving the biological structure of radiomic data. In this way, STFT becomes a critical tool in modern medical diagnosis.

Radiomics offers a powerful approach for quantitatively measuring numerous biological parameters from statistical, morphological, and textural perspectives. However, a key limitation is that it may lead to the omission of variations hidden in the data. For instance, the biophysical structure of cancer tissue, changes in and around the tumor, and the distribution of necrotic areas are generally consistent with complex, multi-component frequency data. Therefore, representing radiomic data solely as statistical or one-dimensional data can fail to capture the full complexity of the biological structure.

Time-frequency analysis reveals the characteristics of cancerous structures and enables more precise identification of tumor-related changes. However, the integrated use of STFT with radiomic data is quite limited in the literature [11]. In most current studies, the STFT method is applied to biomedical signals such as electroencephalography (EEG), electrocardiography (ECG), and phonocardiogram signals, yet a systematic, methodological approach to time-frequency domain re-representation of radiomic-based feature sequences has not been presented. There are several important reasons for this. This situation arises because radiomic data from cancer images exhibit significant variability in size, scale, and heterogeneity, necessitating appropriate windowing strategies during time-frequency transformations. This situation reveals a lack of strong representation of the frequency content of radiomic data and clearly indicates the need for an STFT-based approach. To address this, transforming radiomic features into a two-dimensional time-frequency map via STFT provides a new representation for a better understanding of the cancerous structure. Furthermore, the STFT transformation of radiomics features yields a rich visual representation that deep learning models can process with high accuracy [12]. Therefore, integrating this transformation into clinical decision support systems has significant potential for improving the diagnostic performance of radiomic analyses.

In the current literature, radiomic features are mostly evaluated using statistical methods, which do not adequately capture the frequency components of the cancerous region. Therefore, developing alternative approaches to represent the biological structure in radiomic data while preserving it is of great importance. In this study, radiomic features for different cancer types were converted into slice-position–frequency images using the STFT. A deep learning model was developed to classify three types of lung cancer. Although many models have been proposed in the literature, this study aimed to develop a compact architecture with a small number of trainable parameters and efficient model training. After the model completed classification, the SWAM method was developed to explain the regions of the ordered slice sequence that contributed most strongly to the model’s decision. Using SWAM, decision-relevant slice ranges were highlighted, providing an exploratory indication of the regions influencing the classification result. This method may support the interpretation of model decisions and provide a basis for future studies involving slice-level annotations. In this respect, the study proposes a methodological approach for radiomic analysis.

## 2. Materials and Methods

**Figure 1:**
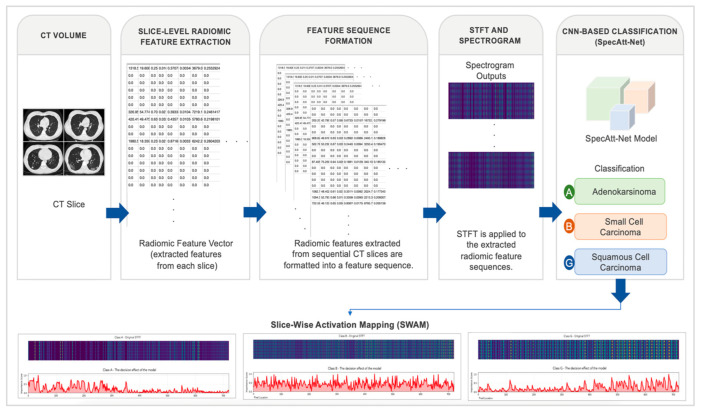
Overview of the proposed CT-based SpecAtt-Net.

This study first examined CT images obtained from lung cancer patients in the Lung-PET-CT-Dx dataset [13]. CT data consists of layers called slices, and each CT can contain a different number of slices. Slice-level radiomic features were extracted from each patient’s CT scans in the dataset. After cleaning and organizing the resulting radiomic dataset, an STFT was applied to represent the signals in the slice-position–frequency domain, and the resulting spectrograms were visually recorded. These visualizations were then fed as input to the proposed deep learning-based model to classify the cancers. To explain the classification process, a slice-based activation mapping method was developed and integrated into the system. The steps of the process are summarized in Figure 1.

### 2.1. Dataset: Lung-PET-CT-Dx

**Figure 2:**
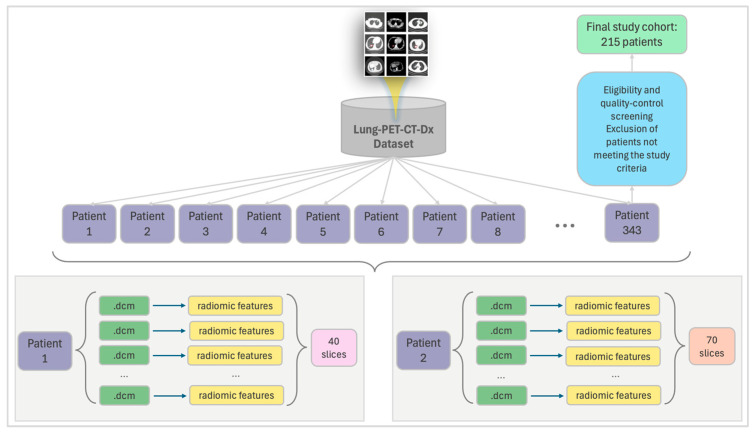
The structure of the dataset used and the processing steps applied.

The dataset used in this study is the open-source Lung-PET-CT-Dx, which contains CT data from patients with lung cancer. These CT images provide a suitable basis for STFT-based radiomic analysis because they are slice-distributed and contain three different cancer types. In the Lung-PET-CT-Dx dataset, each patient’s CT scan contains a different number of slices. For example, one patient’s CT scan may contain 40 slices, while another patient’s scan may contain 70 (Figure 2).

Since CT slices are acquired in an anatomically sequential manner, their increasing, consecutive order plays a crucial role in preserving the tumor’s three-dimensional structural representation. In the context of cancer characterization, this slice continuity is important because tumor morphology, spatial heterogeneity, and lesion extent are represented across successive slices rather than in isolated images. Therefore, the original slice order must not be disrupted, shuffled, or randomly rearranged, as doing so may alter the spatial relationship between slices and compromise the anatomical consistency required for reliable radiomic analysis. Accordingly, the original sequential order of the slices was strictly preserved in this study. In this study, Adenocarcinoma is represented by the letter “A”, Small Cell Carcinoma by the letter “B”, and Squamous Cell Carcinoma by the letter “G”. These cancer types are subsequently referred to as A, B, and G.

The initial dataset was screened according to predefined data-quality criteria before radiomic feature extraction and model development. CT acquisitions containing fewer than 50 slices, corrupted or unreadable files, and cases with incomplete or insufficient imaging information were excluded. Tumor subtypes with an insufficient number of eligible patients to support reliable stratified five-fold evaluation were also omitted from the final analysis. Following these exclusions, the study population comprised 215 patients: 123 in Class A, 36 in Class B, and 56 in Class G. Although stratified patient-level cross-validation and fold-specific class weighting were applied to mitigate class imbalance, the smaller numbers of Class B and Class G cases may still increase the variability of the corresponding class-wise performance estimates.

### 2.2. Radiomic Feature Extraction

Radiomic features were extracted from each CT slice using PyRadiomics version 3.0.1 with a whole-slice binary mask covering the complete image area. The extractor was initialized without an external parameter file, and the default PyRadiomics configuration was retained. Accordingly, fixed-bin-width discretization was performed using a bin width of 25, while additional intensity normalization and image resampling were not applied. The original image spacing was therefore preserved during feature extraction. All available image types and feature classes were enabled, and non-numeric diagnostic outputs were excluded before the subsequent feature-selection and STFT transformation stages. PyRadiomics generated quantitative features describing the intensity distribution, texture, and spatial characteristics of each complete CT slice.

**Figure 3:**
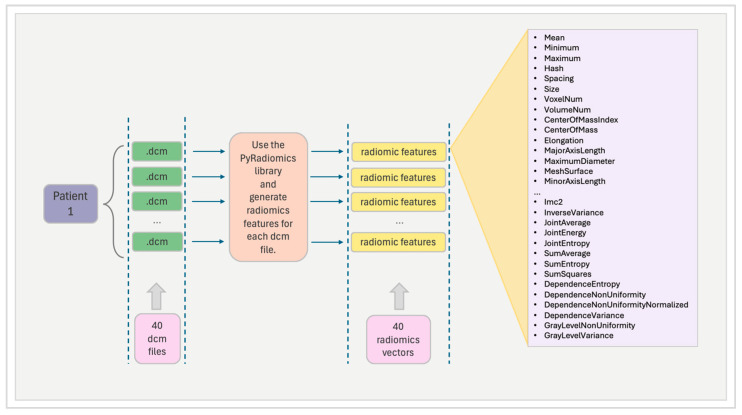
The process of obtaining radiomic data.

About 150 radiomic features were generated for each CT slice, including first-order statistics, the gray-level co-occurrence matrix (GLCM), the gray-level run-length matrix (GLRLM), and the gray-level dependency matrix (GLDM) (Figure 3). Most radiomic features implemented in PyRadiomics comply with the feature definitions established by the Image Biomarker Standardisation Initiative (IBSI), while implementation-specific differences for particular features are documented in the official PyRadiomics documentation [14].

These data were then combined to form a radiomic dataset for each patient. Radiomic analyses in the current literature are typically performed slice-by-slice, with each slice treated as an independent sample. However, examining each slice independently can miss important anatomical continuity and spatial relationships between tumor regions. For example, if a cancerous structure is present between slices 9 and 12, biologically significant connections between consecutive cancerous slices may be lost when the slices are analyzed separately.

### 2.3. Short-Time Fourier Transform (STFT)-Based Transformation

STFT extracts the signal’s frequency components in different segments by analyzing the signal with local windows [15]. Although STFT is widely used in biomedical signal processing, there is no comprehensive study on visualizing radiomic data with STFT and converting it into 2D images. The reason for choosing STFT in this study is its ability to spectrally decompose local variations caused by slice continuity in radiomic arrays and to produce a visual slice-position–frequency map that deep learning models can directly analyze.

$$STFT\left\{x\left(t\right)\right\}\left(\tau ,f\right)=\int_{\left\{-\infty \right\}}^{\left\{\infty \right\}}x\left(t\right)w\left(t-\tau \right)e^{\left\{-j2\pi ft\right\}}dt$$

- x(t): Signal in the time domain;
- w(t − τ): Time window;
- f: Frequency;
- τ: Window position.

STFT transformations were performed in the Python environment using the STFT function of the SciPy [16] library. The Matplotlib library was used for visualizing the slice-position components. The visualizations were created in a completely model-oriented image format by removing axes, titles, and margins.

### 2.4. Determining STFT Parameters

During the STFT transformation of radiomic data, the window length (nperseg) and overlap amount (noverlap) are important hyperparameters that affect the frequency-time resolution of the signal. In this study, the STFT was applied with nperseg = 8 and noverlap = 4, following the structure suggested in the literature for sequences with different signal lengths [17]. The nperseg parameter captures local variations in the radiomic sequence with high time resolution. The 50% overlap ratio (noverlap = 4) maintains continuity between successive STFT windows, preventing the spectral loss of changes shown by the tumor along the slice. Thus, the clarity of small local frequency components is increased, and the slice-based structure of radiomic data is transferred to the slice-position–frequency domain. The STFT outputs obtained with these parameters were then converted to normalized slice-position–frequency images of size 720 × 288 pixels and used as input to the deep learning model.

### 2.5. Handling Radiomic Data in Signal Form

Due to the three-dimensional nature of CT data, processing volumetric images directly with deep learning models may require substantial computational resources and training time [18]. Independent evaluation of radiomic features on a slice basis may also lead to the loss of information related to anatomical continuity and heterogeneity across consecutive slices. In this study, the slice-level radiomic features of each CT acquisition were reconstructed as an anatomically ordered spatial sequence. The radiomic vectors extracted from individual slices were arranged according to their original anatomical order, yielding an acquisition-specific ordered radiomic sequence. Due to missing or incomplete Extensible Markup Language (XML) annotation files, consistent tumour-specific segmentation masks were unavailable for a substantial proportion of the CT acquisitions. Therefore, a binary mask covering the entire two-dimensional CT slice was generated and supplied to PyRadiomics together with the corresponding image. Consequently, the extracted features represented whole-slice intensity and texture characteristics rather than tumour-exclusive properties. These whole-slice radiomic features were used to identify imaging patterns associated with the three lung cancer subtypes and to examine their variation across anatomically consecutive CT slices.

Approximately 150 radiomic features were initially extracted from each eligible CT slice. Patient identifiers, diagnostic metadata, software-version information, library information, and other non-feature entries were excluded using predefined data-cleaning rules. To reduce dimensionality and computational complexity, 84 features were retained through supervised feature selection.

The complete cohort of 215 patients was partitioned at the patient level using stratified five-fold cross-validation. Each CT acquisition was processed independently as an imaging unit; however, cross-validation partitioning was performed at the patient level. When multiple CT acquisitions were available for the same patient, all acquisitions belonging to that patient were collectively assigned to the same cross-validation fold. Consequently, during each iteration, a patient and all associated CT acquisitions were included in their entirety in either the training subset or the validation subset, and no acquisitions from the same patient were distributed between the two subsets. This patient-level grouping prevented information leakage between the training and validation data.

SelectKBest was fitted exclusively to the radiomic features and class labels of the training patients in the corresponding fold. This filter-based univariate feature-selection method evaluates each feature independently according to the strength of its statistical relationship with the target class. The default classification scoring function (f_classif) was used to rank the numerical radiomic features, and the 84 highest-scoring features were retained. The selected feature set was then applied unchanged to the corresponding validation patients without refitting the selector. Class weights were also recalculated independently in each iteration using only the class distribution of the training patients. Thus, no validation patient contributed to feature ranking, feature selection, class-weight calculation, or model training. Each patient was included in the validation subset exactly once across the five iterations, and no separate held-out test set was used for the primary cross-validation analysis.

**Figure 4:**
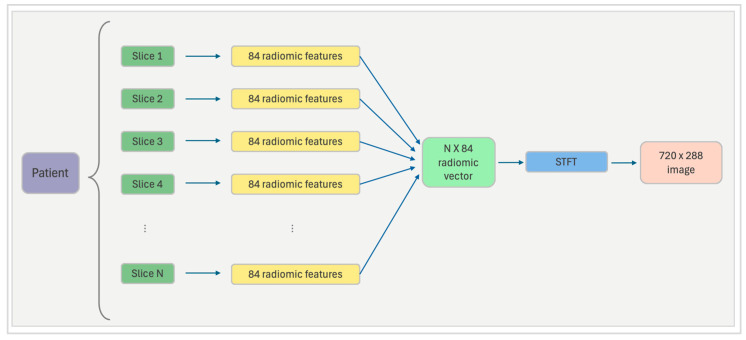
Data preprocessing steps.

For each CT, the selected radiomic features were transformed into an STFT representation. For example, a CT acquisition containing 40 slices produced 40 slice-level vectors, each containing 84 selected radiomic features. These vectors were arranged according to their anatomical slice order to form a 40 × 84 matrix. Similarly, an acquisition containing 100 slices produced a 100 × 84 matrix (Figure 4). When a patient had multiple CT acquisitions, each acquisition was processed independently and represented by a separate radiomic matrix and STFT image.

This method preserves the anatomical ordering of the CT slices, allowing the resulting sequence to reflect spatial variations across consecutive slices. The value *N* represents the number of slices in the CT acquisition for each patient. One contribution of this study is the use of STFT to process feature sequences of different lengths originating from CT acquisitions with variable numbers of slices. Since each acquisition may contain a different number of CT slices, the resulting slice-level radiomic sequences do not have a fixed length, which complicates their direct use in image-based classification models. By applying STFT, these variable-length ordered sequences were converted into fixed-size 720 × 288 image-like representations. This transformation provides a standardized two-dimensional structure while retaining localized variation along the anatomically ordered sequence. Therefore, the proposed STFT-based approach converts variable-length CT slice information into a consistent image format suitable for radiomic analysis and classification. This strategy preserves the spatial ordering of the slices, provides a continuous patient-specific radiomic representation, and offers an alternative to direct three-dimensional image processing. At this stage, the radiomic data were represented as an ordered sequence for slice-position–frequency analysis and prepared for STFT.

**Figure 5:**
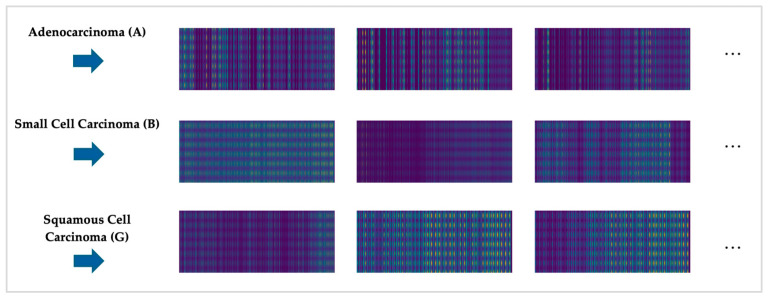
Images obtained by applying STFT to coupled radiomics.

During STFT, the radiomic data from each CT acquisition were processed as a complete ordered sequence. A window length of 8 and an overlap of 4 were used, corresponding to 50% overlap between adjacent windows. The resulting coefficient matrices were rendered as two-dimensional visual representations and resized to 720 × 288 pixels using the same procedure for all acquisitions (Figure 5).

For each CT acquisition, eligible slices were arranged according to their anatomical order, and each slice was represented using the same fixed and consistently ordered set of 84 radiomic features. The slice-level feature vectors were concatenated using a slice-major arrangement. Thus, for an acquisition containing *N* slices, the initial *N* × 84 radiomic matrix was reshaped into a one-dimensional sequence of length 84N, with each consecutive block of 84 values representing one anatomically ordered CT slice. The STFT was then used to encode localized variations in the radiomic profile along this ordered sequence. Because the STFT operates on the concatenated feature sequence and the resulting representation is subsequently resized, positions in the final image correspond to mapped regions of the ordered sequence rather than exact individual CT slices.

One STFT image was generated for each CT acquisition. When multiple acquisitions were available for the same patient, each acquisition was processed separately; however, all STFT images derived from that patient’s acquisitions were collectively assigned to the same cross-validation fold.

### 2.6. Proposed Deep Learning Model: SpecAtt-Net

**Figure 6:**
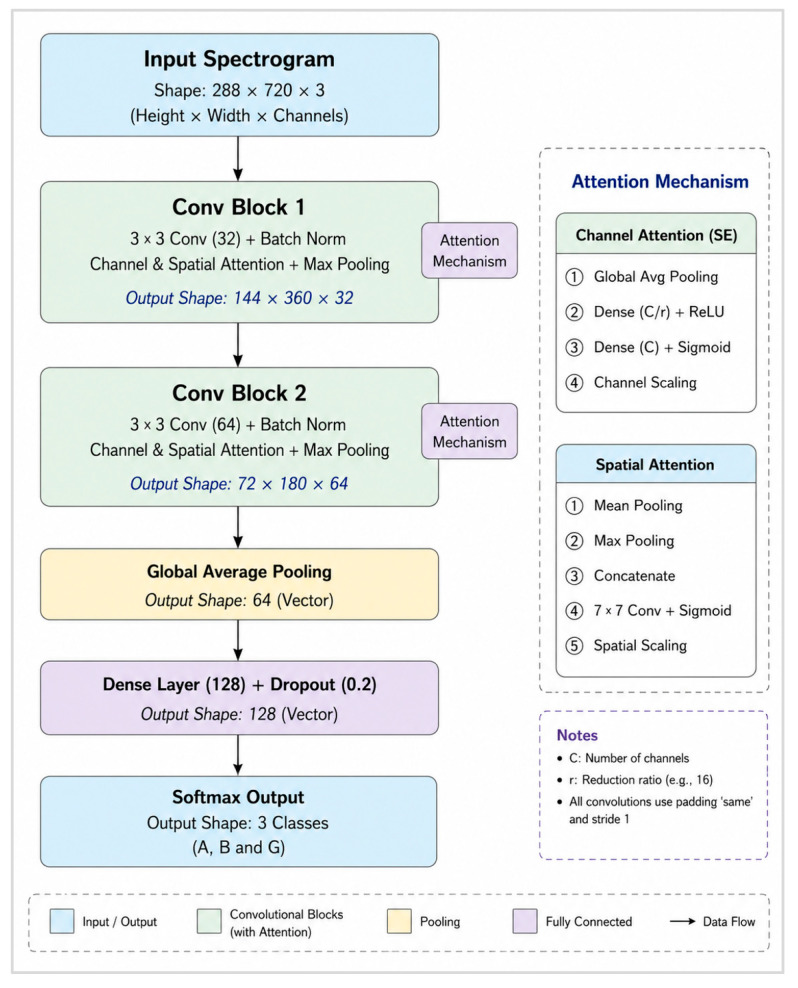
Proposed deep learning model architecture for medical STFT analysis.

In this study, a novel model was developed for lung cancer subtype classification and compared with established deep learning architectures in the literature. One of the main components of the proposed model is the sequential dual-attention mechanism applied after each convolutional layer (Figure 6). First, the Squeeze-and-Excitation (SE) module learns inter-channel relationships by aggregating global information during the squeeze operation and recalibrating informative feature channels during the excitation operation. This enables the model to emphasize channels containing discriminative spectral information and reduce the contribution of noisy or less relevant channels. Subsequently, the spatial-attention module generates channel-wise average and maximum feature maps, combines them, and applies a 7 × 7 convolution with sigmoid activation to identify informative spatial regions within the STFT representation. Thus, the combined channel and spatial attention mechanism enables SpecAtt-Net to refine feature representations across both channel and spatial dimensions.

The L2 Regularization [19], Batch Normalization, and Dropout techniques used in the model are important factors that help prevent overfitting. L2 regularization prevents weights from becoming too large, improving generalization. Batch Normalization was applied after each convolutional layer to stabilize the distribution of intermediate activations and support faster and more stable model training. In addition, a Dropout layer was included before the final classification layer to reduce co-adaptation among neurons during training. At the end of the architecture, Global Average Pooling was used instead of the conventional Flatten operation. This substantially reduced the number of trainable parameters and computational requirements while improving the model’s robustness to spatial variations.

To mitigate the effect of class imbalance, a class-weighted training strategy was employed. Class weights were calculated independently using the training subset of each cross-validation fold according to a balanced weighting scheme. Underrepresented classes were assigned higher weights, whereas classes with larger numbers of samples received lower weights. These weights were applied during the calculation of categorical cross-entropy loss, thereby increasing the penalty associated with misclassifying minority-class samples. Furthermore, stratified five-fold cross-validation was used to preserve the original class distribution as closely as possible across the training and validation subsets. The combined use of class-weighted learning and stratified cross-validation reduced the model’s tendency to favor the majority class without altering the original dataset through oversampling or synthetic sample generation.

### 2.7. Implementation and Reproducibility Details

All experiments were implemented in Python 3.8.18 on a macOS 15.6.1 ARM64 environment. SpecAtt-Net was developed and trained using TensorFlow 2.13.0 and Keras 2.13.1. PyRadiomics 3.0.1 and SimpleITK 2.3.1 were used for radiomic feature extraction and image processing, respectively. STFT computation and visualization were performed using SciPy 1.10.1 and Matplotlib 3.7.5. OpenCV 4.10.0 was used for image loading and resizing, while NumPy 1.24.3, pandas 2.0.3, scikit-learn 1.3.2, and Seaborn 0.13.2 were used for numerical processing, data organization, evaluation, and visualization. The experiments were conducted on an Apple MacBook Pro equipped with an Apple M3 Pro processor and 18 GB of unified memory, with GPU acceleration enabled through TensorFlow.

The STFT images were resized to a spatial resolution of 720 × 288 pixels using INTER_AREA interpolation, retained as three-channel inputs, and normalized to the [0, 1] range. Stratified five-fold cross-validation was performed with shuffling and a random state of 42. The model was trained using the Adam optimizer with an initial learning rate of 1 × 10^−4^, categorical cross-entropy loss, a batch size of 8, and a maximum of 300 epochs. L2 regularization with a coefficient of 0.001 and dropout with a rate of 0.2 were applied. Class weights were calculated separately from the training subset of each fold using a balanced weighting scheme. The learning rate was reduced by a factor of 0.5 when the validation loss did not improve for 10 consecutive epochs, with a minimum learning rate of 1 × 10^−6^. Early stopping monitored validation accuracy with a patience of 50 epochs, and the best-performing weights were restored.

## 3. Results

This section presents the experimental findings obtained from the five-fold cross-validation analysis of SpecAtt-Net. First, the fold-specific classification results are reported in terms of accuracy, precision, recall, and F1-score for each class. Subsequently, the overall out-of-fold performance, ablation analysis, learning curves, and comparisons with the evaluated reference architectures are presented.

Table 1 presents the fold-wise classification performance of SpecAtt-Net. Fold 1 achieved an accuracy of 93.02% and demonstrated classification performance across all three classes. Class A showed high precision and recall, while Class B exhibited balanced precision and recall values. Class G achieved high recall and precision, indicating that all Class G samples were correctly identified, although a small number of samples from the other classes were incorrectly assigned to this class. Fold 2 produced the lowest accuracy at 81.40%, and represented the most challenging validation subset. Class A achieved high precision but substantially lower recall, indicating that although all predictions assigned to Class A were correct, several actual Class A samples were classified as other classes. Class B also showed reduced recall. Class G achieved perfect recall but considerably lower precision, indicating that all Class G samples were identified while several samples from the other classes were incorrectly predicted as Class G. Fold 3 achieved an accuracy of 86.05%. Class A showed high precision but lower recall, indicating that some Class A samples were missed. Class B exhibited relatively high recall but lower precision, whereas Class G showed moderate precision and high recall. These findings indicate that class discrimination was more difficult in Fold 3 than in Folds 1, 4, and 5. Fold 4 achieved the highest classification performance, with an accuracy of 97.67%. Class B achieved precision, recall, and F1-score values of 1.0000, indicating that all Class B samples in this fold were correctly classified. Class A achieved perfect precision and high recall, indicating that only one actual Class A sample was missed. Class G achieved perfect recall and high precision, indicating that all Class G samples were detected, with only a small number of false-positive predictions. Overall, only one of the 43 validation samples was misclassified in this fold. Fold 5 also demonstrated strong classification performance, achieving an accuracy of 95.35%. Class A showed high and balanced precision and recall. Class B achieved perfect precision but slightly lower recall, indicating that one actual Class B sample was missed while no samples from the other classes were incorrectly predicted as Class B. Class G achieved perfect recall and high precision, showing that all Class G samples were detected with a limited number of false-positive predictions. Across the five folds, SpecAtt-Net achieved a mean accuracy of 90.70 ± 6.78% and a mean macro F1-score of 89.45 ± 7.70%. Class A showed the highest mean precision, whereas Class G generally achieved the highest recall but comparatively lower precision, particularly in Fold 2. Class B exhibited greater variation in both precision and recall across the validation folds. Fold-wise accuracy ranged from 81.40% to 97.67%, indicating noticeable variability across the validation subsets. Therefore, the proposed model demonstrated promising classification performance, although further evaluation using larger, more balanced, and independent external cohorts is required to confirm its generalizability.

To provide a controlled architecture-level comparison, an additional experiment was conducted in which SpecAtt-Net and the reference CNN models were trained from random initialization using the same patient-level five-fold cross-validation protocol. This comparison was performed as a separate training run; therefore, the SpecAtt-Net performance reported in Table 2 differs from the final-model results presented in Table 1 due to stochastic variation between independent runs. Results obtained within the same comparison run were used consistently for all models.

Table 2 compares SpecAtt-Net with VGG16, ResNet50, and MobileNetV2 under the same random-initialization and five-fold cross-validation protocol. SpecAtt-Net achieved the highest mean accuracy of 86.98 ± 8.16% and the highest macro F1-score of 84.39 ± 9.78%. It also obtained higher macro precision and macro recall than the evaluated reference architectures. Despite having only 29,231 trainable parameters, SpecAtt-Net provided a favorable balance between classification performance and parameter efficiency when all models were trained without external pretraining.

Table 3 presents paired statistical comparisons between SpecAtt-Net and the reference CNN architectures using pooled out-of-fold predictions from the same patient-level five-fold partitions. The accuracy and macro F1-score differences are reported in percentage points as SpecAtt-Net minus the corresponding reference model, together with their 95% confidence intervals (CIs). Positive differences and confidence intervals entirely above zero indicate better performance for SpecAtt-Net. Compared with VGG16, SpecAtt-Net improved accuracy by 22.33 percentage points and macro F1-score by 33.40 percentage points. Larger differences were observed compared to ResNet50 and MobileNetV2. McNemar’s exact test compared paired correct and incorrect predictions for the same samples, while Holm adjustment accounted for multiple comparisons. All adjusted *p*-values were below 0.001, indicating significantly higher paired classification accuracy for SpecAtt-Net. Unlike Table 2, which reports mean and standard deviation across folds, Table 3 evaluates paired differences using pooled out-of-fold predictions.

**Figure 7:**
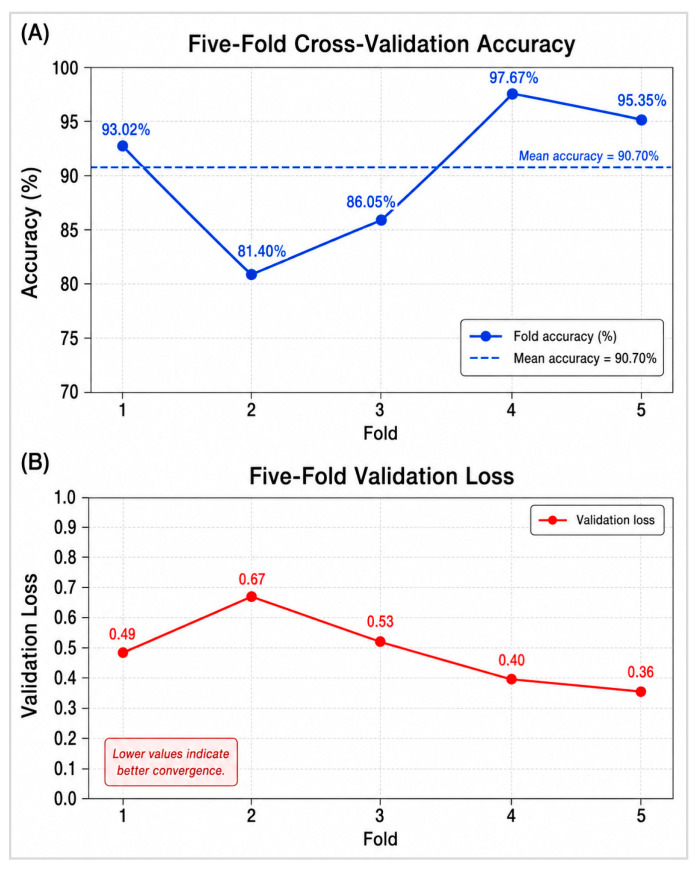
Five-fold cross-validation accuracy and validation loss of proposed SpecAtt-Net model: (**A**) fold-wise validation accuracy and mean accuracy; (**B**) fold-wise validation loss.

Figure 7 presents the fold-wise accuracy and validation loss values obtained during five-fold cross-validation. The model achieved accuracy values of 93.02%, 81.40%, 86.05%, 97.67%, and 95.35% across Folds 1–5, respectively, corresponding to a mean accuracy of 90.70%. Although some variability was observed between folds, particularly in Fold 2, the model achieved an accuracy above 86% in four of the five folds. The corresponding validation loss values ranged from 0.36 to 0.67. Fold 2 exhibited the highest validation loss and the lowest accuracy, whereas Folds 4 and 5 showed lower loss values and higher classification performance. Overall, the inverse relationship between validation loss and accuracy across the folds supports the consistency of the reported cross-validation results while also indicating fold-dependent variation in model generalization.

**Figure 8:**
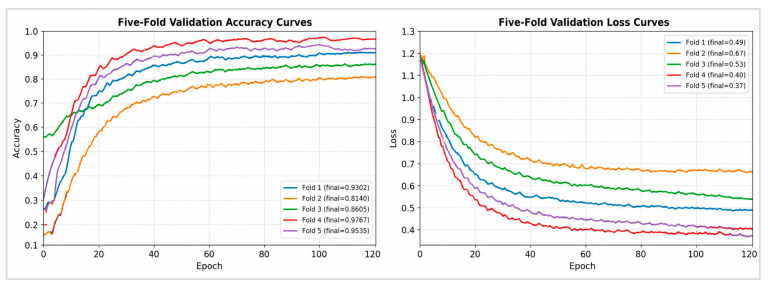
Five-fold validation accuracy and loss curves of the proposed SpecAtt-Net model.

Figure 8 shows the fold-wise validation accuracy and loss curves of SpecAtt-Net during five-fold cross-validation. Overall, the accuracy increases and the loss decreases consistently, indicating stable learning behavior and reasonable generalization across folds.

Table 4 presents the overall class-wise performance obtained by pooling the out-of-fold predictions from all five cross-validation folds. Class A achieved the highest precision at 98.20%, indicating that predictions assigned to this class were rarely false positives. Its recall of 88.62% shows that some actual Class A cases were assigned to other classes; nevertheless, the resulting F1-score of 93.16% indicates a strong balance between precision and recall. Class B achieved equal precision and recall values of 86.11%, resulting in an F1-score of 86.11% and indicating comparatively balanced classification performance for this class. Class G achieved the highest recall at 98.21%, demonstrating that nearly all Squamous Cell Carcinoma cases were correctly identified. However, its lower precision of 80.88% indicates that some samples from the other classes were incorrectly classified as Class G. The macro-averaged precision, recall, and F1-score were 88.40%, 90.98%, and 89.33%, respectively. These results demonstrate strong overall class discrimination, although the comparatively lower precision for Class G and the variability observed across the individual cross-validation folds should be considered when interpreting the model’s generalization performance.

Table 5 presents the ablation analysis conducted to evaluate the contribution of the Squeeze-and-Excitation (SE) channel-attention module in SpecAtt-Net. All other architectural components, including the spatial-attention module and the training settings, were retained unchanged. The full dual-attention configuration achieved an accuracy of 90.70 ± 6.78% and a macro F1-score of 89.44 ± 7.70%. After removing the SE module, the accuracy decreased to 86.98 ± 6.28%, while the macro F1-score decreased to 84.33 ± 6.95%.

Table 6 presents a separate experiment conducted to evaluate the effect of the STFT input-channel configuration. The same STFT coefficient representations were rendered using two different input formats. In the Viridis-mapped RGB configuration, the STFT values were mapped to the Viridis colour scale and represented as three-channel images. In the grayscale configuration, the same representations were converted into single-channel images. The resulting images were then used as inputs to the corresponding SpecAtt-Net configurations. The Viridis-mapped RGB input achieved a mean fold accuracy of 88.84 ± 6.45% and a mean macro F1-score of 86.88 ± 6.82%, whereas the single-channel grayscale input achieved 85.12 ± 5.35% accuracy and 80.71 ± 6.64% macro F1-score. The pooled out-of-fold accuracy and macro F1-score were also higher for the RGB configuration, reaching 88.84% and 87.00%, respectively, compared with 85.12% and 80.98% for the grayscale configuration. These findings support the selection of the three-channel Viridis representation as the input format for the proposed model. This channel-configuration comparison was performed independently from the SE ablation analysis presented in Table 5; therefore, numerical results across the two tables should not be interpreted as outcomes from the same experimental run.

**Figure 9:**
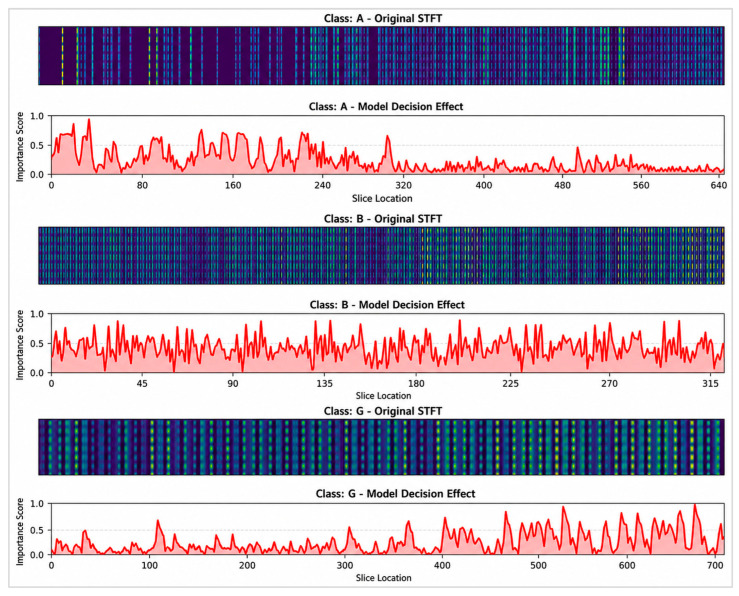
Generation of SWAM importance vectors and analysis of raw activation signals.

Figure 9 visualizes the STFT intervals that influence the classification results by converting them into a graphical representation. For each class, higher values indicate intervals that contributed more strongly to the model’s decision. This visualization forms the basis of the SWAM approach proposed in this study and provides an exploratory interpretation of the decision-relevant regions.

**Figure 10:**
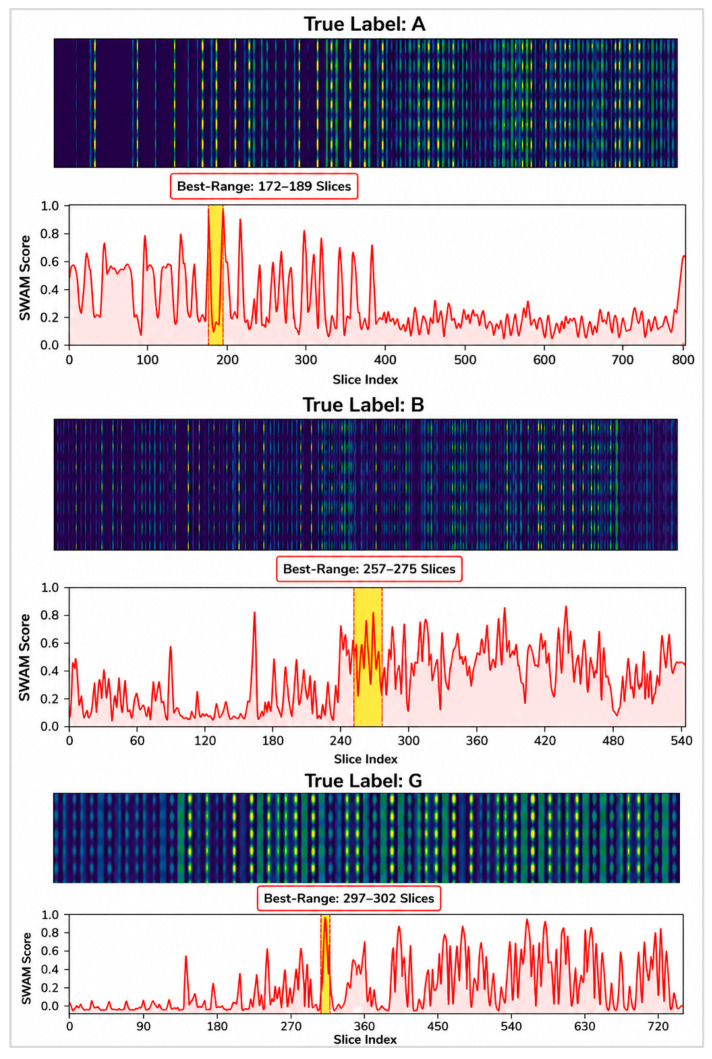
Dynamic thresholding and pathological site localization in SWAM analysis.

The interpretability of the proposed SpecAtt-Net model was examined through a dedicated post hoc analysis designed to relate global image-level classification decisions to decision-relevant regions of the ordered slice sequence. Given that the input radiomic features are synthesized from successive CT slices and converted into a 720 × 288 STFT representation, traditional two-dimensional saliency methods may produce spatially dispersed activation patterns that are difficult to interpret in relation to the original slice sequence. To provide a more sequence-oriented representation, a methodological adaptation called Slice-Wise Activation Mapping (SWAM) was implemented (Figure 10). This method transforms the standard two-dimensional activation representation into a one-dimensional importance distribution corresponding to mapped positions along the ordered slice sequence.

The interpretability of the proposed SpecAtt-Net architecture was examined through the SWAM method, which visualizes the model’s decision-making process across the mapped slice-sequence representation. By extracting gradient-based activation maps from the final convolutional layer, a one-dimensional significance vector was generated, quantifying the contribution of mapped slice-sequence regions to the classification decision. Our findings showed that the SWAM curves consistently peaked at specific slice intervals where radiomic feature fluctuations were most pronounced, suggesting that the model focused on regions containing potentially relevant radiomic variations. The developed dynamic thresholding mechanism identified the “Best-Range” by determining the most significant segment of the ordered slice sequence. In correctly classified cases, the identified ranges provided an exploratory indication of the regions that contributed most strongly to the model’s decision. Because no formal slice-level reference standard or quantitative overlap analysis was included, these findings should not be interpreted as validated anomaly localisation. This weakly supervised importance analysis, achieved without slice-level ground-truth labels, supports the interpretability of the feature extraction and classification process.

## 4. Discussion

This study investigated the potential of spectral representations derived from slice-level radiomic features in lung cancer classification using CT images. The primary motivation stemmed from the limitations of traditional radiomic and deep learning approaches in volumetric medical imaging, where anatomical structures are represented as a three-dimensional volume composed of consecutive two-dimensional CT slices. These limitations include the different amounts of radiomic data, the limited interpretability of black-box models, and the insufficient modeling of sequential dependencies between successive slices. This study aims to develop a novel, explainable classification approach that captures both spectral tissue patterns and spatial transitions across CT slices, while providing evidence on the slice groups that contribute most to the model’s decision-making. For this purpose, SpecAtt-Net, a dual-attention-based architecture, was proposed to highlight distinctive radiomic patterns by transforming anatomically ordered slice-level radiomic data into STFT-based position–frequency representations while preserving the original inter-slice spatial sequence. In addition to this architecture, an interpretability approach called Slice-Wise Activation Mapping has been developed due to the absence of slice-level labels in clinical datasets. With this approach, two-dimensional activation responses are converted into one-dimensional slice significance vectors, providing an exploratory indication of the slice-sequence intervals that contribute most strongly to the classification decision. In this context, the study findings were compared with existing architectures reported in the literature, focusing on classification performance metrics.

In lung cancer studies, a significant portion of radiomic applications traditionally relies on feature extraction from 3D tumor volumes because tumors are heterogeneous. Furthermore, 2D approaches are considered an alternative in clinical practice due to segmentation costs, differences in measurement standards across different centers, and variations in data quality. 2D radiomics can sometimes match or exceed 3D radiomics in predictive performance. Plus, 2D features are often easier and cheaper to use [20,21]. Therefore, treating slice-level extracted radiomic features as a sequence can provide a representation that captures tumor heterogeneity. To convert the high-dimensional feature vectors generated for each slice with slice-level feature extraction into a “patient/tumor” decision, either statistical combinations such as mean, median, percentiles, max-min, or modeling strategies that preserve the sequential structure are required.

Considering the variation in radiomic patterns across anatomically consecutive slices as an ordered signal enables these changes to be represented in a slice-position–frequency space. Slice-level radiomic feature vectors were therefore concatenated according to their original anatomical order and transformed using STFT to generate a spectral representation of local radiomic variations across slice positions. This approach combines quantitative radiomic feature extraction with signal-processing-based position–frequency analysis, thereby making subtype-associated spatial variation patterns more distinguishable.

Spectrograms generated with the STFT visualize which frequency components are dominant in specific intervals within sequences that carry meaningful patterns in non-stationary or local windows [22,23]. It is known that STFT-based spectrograms are becoming widespread in the medical field, especially for biosignal classification, by converting 1D time series into 2D image representations using STFT and leveraging the power of visual deep learning architectures such as Convolutional Neural Networks (CNNs) [18]. There are also examples of frequency-based approaches being used in lung image analysis for problems such as nodule characterization and tissue discrimination. This trend supports the idea that anatomically ordered radiomic feature sequences can also be represented using signal-processing methods. Tissue changes along slices can produce a heterogeneity pattern at a specific frequency range. This situation allows classifiers to learn distinctive representations.

Therefore, converting radiomic features into an image-like representation using STFT is not only a technical transformation but also changes the modeling structure. In classical radiomic studies, features are mostly tabular in nature and are often used with machine learning algorithms such as Support Vector Machine (SVM) [24], Random Forest [25], and XGBoost [26]. Generating images from radiomics, however, allows 2D representations such as spectrograms to be naturally processed by CNNs. This point offers advantages in pattern learning, representation extraction, and data enhancement. Studies showing that radiomic-based models can be used in histological subtype differentiation and similar classification tasks in lung cancer reveal that the field directly addresses clinically significant problems.

A review of the literature (Table 7) reveals that while extracting radiomic features from lung cancer CT data offers significant contributions to diagnostic and classification problems, the modeling and representation of these features are not yet sufficiently mature. In particular, the simplification of slice-level radiomic information, largely through statistical summarization, may lead to the loss of structural and sequential information across anatomically ordered slices, while the predominant use of tabular radiomic data limits the potential of deep learning-based representation learning. Although STFT- and spectrogram-based deep learning approaches have been applied to physiological signals such as ECG, EEG, and respiratory sound signals, no directly comparable approach applying STFT to anatomically ordered CT radiomic features has been systematically reported. This study aims to address this representation and modeling gap by treating slice-level radiomic features as an anatomically ordered spatial sequence, converting them into slice-position–frequency representations using STFT, and using the resulting images for lung cancer subtype classification.

Based on studies in the literature, the results obtained from this study support the idea that radiomic features obtained from lung cancer CT images can be represented using slice-position–frequency analysis to generate image representations, and successful lung cancer subtype classification can be performed using these representations. The results reveal that treating radiomic data not only as static statistical features but also as an anatomically ordered spatial signal may provide additional discriminative information. The vast majority of studies in the current literature on lung cancer classification rely on directly inputting radiomic features into machine learning algorithms or deep learning-based classifiers. The methodology developed in this study reveals the diagnostic potential of converting radiological data into high-dimensional radiomic spectrograms and analyzing these data with SpecAtt-Net. While traditional approaches in the literature generally focus on raw pixels or static radiomic features, the sequential spatial arrangement of radiomic features across anatomically consecutive slices used in this study enables simultaneous modeling of inter-slice anatomical continuity and textural heterogeneity. This structural arrangement may have contributed to the model’s discriminative performance by allowing it to learn not only the textural signatures captured by the convolutional layers but also the variations in these signatures across consecutive slices.

The SpecAtt-Net approach, central to the model’s decision-making, played a critical role in selecting distinctive radiomic patterns from a high-dimensional feature space. One of the most important points to discuss is how the model extracts specific textural patterns from noisy cancerous tissue data. The integration of channel and spatial attention blocks enabled the model to suppress less relevant regions of the representation and focus on radiomic variations associated with the classification task. This allowed the model not only to achieve high accuracy rates but also to prioritize decision-relevant feature groups.

One of the study’s most original contributions, the SWAM method, provides an exploratory interpretation of the often-criticized “black-box” nature of deep learning models. The significance vectors obtained via SWAM highlighted concentrated regions of the ordered slice sequence that contributed strongly to the model’s classification decisions. These patterns provide an exploratory indication of decision-relevant slice intervals but do not establish the precise location or extent of pathological tissue. Because no formal slice-level reference standard or quantitative overlap analysis was included, the findings should not be interpreted as validated localisation of abnormalities. This weakly supervised approach may provide a basis for future studies investigating its potential as a pre-screening or decision-support tool, subject to further validation using radiologist-annotated slice-level references.

Finally, the dynamic thresholding and widest-range selection logic used in SWAM was designed to reduce isolated importance peaks and identify a continuous decision-relevant range. The identified range may provide clinicians with additional visual evidence regarding the slice-sequence segment that contributed most to the model’s decision, thereby supporting the interpretation of the classification result. This approach offers an exploratory explainability strategy for radiomic analysis and requires further validation before its clinical relevance can be established. The results suggest that the integrated approach can provide interpretable information for complex oncological imaging data. In addition, the compatibility of STFT-based images with deep learning architectures may support their evaluation in multicenter and large-scale datasets.

The dataset’s size and single-center nature may limit the generalizability of the results. Validation studies on different CT devices, imaging protocols, and patient populations will more strongly demonstrate the clinical applicability of the method. Although STFT images have yielded successful classification results, pathological and clinical correlations are needed to interpret the biological equivalents of these representations in more detail. In future studies, associating slice-position–frequency components with specific histopathological features will enhance the method’s translational value.

## 5. Conclusions

This study indicates that converting slice-level radiomic features into STFT-based spectrogram representations and analyzing them with the proposed classification architecture may support classification performance and model interpretability in oncological imaging. The proposed approach addresses an important limitation in volumetric CT analysis by transforming sequential radiomic vectors into structured image-like representations while preserving the ordered relationship between consecutive slices. In this way, the model can capture not only discriminative spectral tissue characteristics but also spatial transitions reflecting radiomic variation across anatomically ordered slices. The proposed SpecAtt-Net model not only classifies complex tissue signatures with high precision but also provides interpretable evidence regarding the slice groups that contribute most strongly to the classification decision. Unlike conventional approaches that often require detailed slice-level annotations, the proposed methodology provides an exploratory indication of decision-relevant slice regions using global patient-level labels. This ability may be relevant to medical imaging applications, where manual slice-by-slice labeling is time-consuming, subjective, and highly dependent on radiologist expertise.

One of the notable exploratory findings of this research is the potential of the best-range data identified by SWAM analysis to support the interpretation of model decisions in future clinical studies. By highlighting the slice groups that contributed most strongly to the classification decision, the proposed approach may assist future investigations into slice prioritization and annotation. Since the model provides decision-relevant evidence based on learned imaging patterns, it may contribute to a more structured interpretation process; however, its effect on radiological variability and clinical workflows has not yet been evaluated.

In addition to its classification and interpretability-related outputs, the proposed model offers practical advantages in terms of model compactness. The average results across the five folds showed that the proposed model achieved higher classification performance than the evaluated randomly initialized reference architectures while using substantially fewer trainable parameters. These properties may support its future evaluation in computationally constrained research settings.

Overall, the findings suggest that artificial intelligence may be used not only for classification but also as an interpretable and computationally efficient component of future medical imaging decision-support systems. By combining lung cancer subtype classification, reduced computational complexity, preservation of inter-slice spatial continuity, and exploratory slice-sequence interpretability, the proposed STFT-based radiomic approach provides a proof-of-concept approach for incorporating explainable artificial intelligence principles into radiological image analysis. However, the slice-level interpretability findings should be regarded as preliminary and require further clinician-reviewed validation.

Owing to its compact architecture and low parameter count, SpecAtt-Net may provide a suitable foundation for future computer-assisted imaging applications and further evaluation in resource-conscious computational settings. Nevertheless, the proposed model should be regarded as a proof-of-concept approach rather than a deployment-ready clinical system. Further architectural development, external multicentre validation, clinician-reviewed interpretability assessment, and prospective workflow studies are required to establish its generalisability and clinical utility.

Future studies will aim to improve the generalisability of the proposed approach by evaluating it across larger multicentre datasets, different cancer types, and alternative imaging modalities. The approach presented in this study provides a methodological basis for further investigation of computationally efficient and interpretable artificial intelligence approaches in personalised medicine and precision oncology.

## Figures and Tables

**Table 1:** Fold-wise classification performance of SpecAtt-Net under five-fold cross-validation.

Fold	Class	Precision	Recall	F1-Score	Accuracy	Time (Seconds per Epoch)
1	A	0.9583	0.9200	0.9388	0.9302	172.0
	B	0.8571	0.8571	0.8571		
	G	0.9167	1.0000	0.9565		
2	A	1.0000	0.7600	0.8636	0.8140	165.5
	B	0.8333	0.7143	0.7692		
	G	0.6111	1.0000	0.7586		
3	A	1.0000	0.8400	0.9130	0.8605	183.0
	B	0.6667	0.8571	0.7500		
	G	0.7692	0.9091	0.8333		
4	A	1.0000	0.9583	0.9787	0.9767	174.5
	B	1.0000	1.0000	1.0000		
	G	0.9167	1.0000	0.9565		
5	A	0.9583	0.9583	0.9583	0.9535	167.0
	B	1.0000	0.8571	0.9231		
	G	0.9231	1.0000	0.9600		

**Table 2:** Comparison of SpecAtt-Net with randomly initialized reference CNN architectures under the same five-fold cross-validation protocol.

Model	Accuracy (%)	Macro Precision (%)	Macro Recall (%)	Macro F1-Score (%)	Trainable Parameters
SpecAtt-Net	86.98 ± 8.16	85.11 ± 9.88	85.32 ± 9.35	84.39 ± 9.78	29,231
VGG16	64.65 ± 6.66	46.96 ± 20.92	52.82 ± 12.05	44.64 ± 12.15	14,780,739
ResNet50	45.12 ± 5.84	32.13 ± 8.43	34.89 ± 3.52	32.14 ± 5.62	23,797,251
MobileNetV2	46.05 ± 10.83	38.38 ± 4.73	37.73 ± 5.39	35.48 ± 4.96	2,388,227

**Table 3:** Paired statistical comparisons of SpecAtt-Net with the reference CNN architectures based on pooled out-of-fold predictions.

Comparison	Accuracy Difference (Percentage Points, 95% CI)	Macro F1-Score Difference (Percentage Points, 95% CI)	Exact McNemar *p*-Value	Holm-Adjusted *p*-Value
SpecAtt-Net vs. VGG16	+22.33 (+14.88, +29.30)	+33.40 (+24.72, +42.03)	<0.001	<0.001
SpecAtt-Net vs. ResNet50	+41.86 (+33.95, +49.77)	+49.64 (+41.01, +57.87)	<0.001	<0.001
SpecAtt-Net vs. MobileNetV2	+40.93 (+33.94, +48.37)	+46.79 (+38.47, +55.19)	<0.001	<0.001

**Table 4:** Overall class-wise discrimination performance of SpecAtt-Net based on pooled out-of-fold predictions across lung cancer types.

Class	Precision (%)	Recall (%)	F1-Score (%)
A	98.20	88.62	93.16
B	86.11	86.11	86.11
G	80.88	98.21	88.71
Macro Average	88.40	90.98	89.33

**Table 5:** Ablation analysis of the Squeeze-and-Excitation channel-attention module in SpecAtt-Net.

Configuration	Accuracy (%)	Macro Precision (%)	Macro Recall (%)	Macro F1-Score (%)
Full dual-attention	90.70 ± 6.78	89.40 ± 7.72	90.88 ± 6.29	89.44 ± 7.70
Without SE attention	86.98 ± 6.28	84.21 ± 7.82	85.79 ± 5.19	84.33 ± 6.95

**Table 6:** Performance comparison of Viridis-mapped RGB and single-channel grayscale STFT representations.

Configuration	Accuracy (%)	Macro Precision (%)	Macro Recall (%)	Macro F1-Score (%)
Viridis-mapped RGB	88.84 ± 6.45	88.58 ± 6.81	87.54 ± 6.08	86.88 ± 6.82
Single-channel grayscale	85.12 ± 5.35	86.66 ± 4.98	81.05 ± 6.09	80.71 ± 6.64

**Table 7:** Representative frequency-domain and spectrogram-based deep learning studies in biomedical data analysis.

No.	Year	Study	Model/Method	Performance
1	2023	Cardiac arrhythmia detection using deep learning approach and time frequency representation of ECG signals [27]	ECG → time-frequency representation (Morse wavelet spectrogram) → fine-tuned ResNet-50/AlexNet CNN	Acc 99.2%, Sens 99.2%, Spec 99.6%
2	2023	EEG driving fatigue detection based on log-Mel spectrogram and convolutional recurrent neural networks [28]	EEG → STFT + log-Mel spectrogram → CNN + Recurrent Neural Network (RNN)	Acc 88.39%, F1 ≈ 86.37%
3	2023	Evaluation and Exploration of Machine Learning and Convolutional Neural Network Classifiers in Detection of Lung Cancer from Microarray Gene—A Paradigm Shift [29]	Short Term Fourier, Particle Swarm Optimization and Harmonic Search → Nonlinear Regression, Gaussian Mixture Model, Softmax Discriminant, Naive Bayes, SVM	Acc 94.47%
4	2024	Spectrogram-Based Arrhythmia Classification Using Three-Channel Deep Learning Model with Feature Fusion [30]	ECG → STFT spectrogram + HOG + Local Binary Pattern → CNN + Gated Recurrent Unit (feature fusion)	Acc ≈ 99.80% (for 3-classes) Acc ≈ 99.76% (for 5-classes)
5	2024	A real-time epilepsy seizure detection approach based on EEG using short-time Fourier transform and Google-Net CNN [31]	EEG → STFT spectrogram → GoogLeNet-based CNN	Acc 97.74%, Sens 98.90%
6	2024	Classification of EEG spectrogram images with deep learning models [32]	EEG → spectrogram images → CNN models (ResNet-50, Xception, custom CNN)	Acc for ResNet50 97%, Acc for Xception 100%, Acc for custom CNN 99%
7	2024	Deep learning and feature fusion-based lung sound recognition model to diagnoses the respiratory diseases [33]	Lung sounds → spectrogram → combined feature vector → CNN base models	Acc 96.03%
8	2025	Arrhythmia classification based on multi-input convolutional neural network with attention mechanism [34]	ECG → STFT + double-entry CNN branches + Squeeze-and-Excitation attention blocks	Dataset 1 Acc 99.13%, Macro-F1 94.46%, Dataset 2 Acc 95.84%, Macro-F1 95.91%
9	2025	Deep Learning-Based Classification and Combined Transform-Based Feature Extraction Approach for Mental Stress Prediction of Human Beings Using EEG [35]	EEG → Short-Term Fourier Transform-Randon Transform → combined model	Dataset 1 Acc 97.8% Dataset 2 Acc 96.3%
10	2025	Deep Learning-Enhanced Spectrogram Analysis for Biomedical Signal Classification [36]	Biomedical signals → time-frequency spectrograms → fine-tuned ResNet50	Acc 93.37%
11	2025	Evaluation of Deep Learning Methods for Pulmonary Disease Classification [37]	Respiratory sounds → STFT features → CNN, RNN, Long Short-Term Memory (LSTM) and hybrid CNN-RNN-LSTM	Acc ≈ 88%
12	2025	Deep Learning-Driven Early Diagnosis of Respiratory Diseases using CNN-RNN Fusion on Lung Sound Data [38]	Lung sounds → spectrogram → CNN	Acc 91.7–94.0%

## Data Availability

The imaging dataset analyzed in this study is publicly available from The Cancer Imaging Archive (TCIA) (Lung-PET-CT-Dx collection, https://www.cancerimagingarchive.net/collection/lung-pet-ct-dx/ (accessed on 29 July 2026)). The radiomic features generated during the present study are available from the corresponding author upon reasonable request.

## Abbreviations

The following abbreviations are used in this manuscript:

CT	Computed tomography
STFT	Short-Time Fourier transform
SWAM	Slice-wise activation mapping
GLCM	The gray-level co-occurrence matrix
GLRLM	The gray-level run-length matrix
GLDM	The gray-level dependency matrix
SE	Squeeze-and-excitation
XAI	Explainable artificial intelligence
EEG	Electroencephalography
ECG	Electrocardiography
XML	Extensible Markup Language
CNNs	Convolutional Neural Networks
RNN	Recurrent Neural Network
LSTM	Long Short-Term Memory
SVM	Support Vector Machine
CI	Confidence Interval
